# Occurrence of clinical mastitis in primiparous Estonian dairy cows in different housing conditions

**DOI:** 10.1186/1751-0147-48-21

**Published:** 2006-11-21

**Authors:** Piret Kalmus, Arvo Viltrop, Birgit Aasmäe, Kalle Kask

**Affiliations:** 1 Department of Therapy, Institute of Veterinary Medicine and Animal Science, Estonian University of Life Sciences, 51014 Tartu, Estonia; 2 Department of Infectious Diseases, Institute of Veterinary Medicine and Animal Science, Estonian University of Life Sciences, 51014 Tartu, Estonia; 3 Department of Animal Environment and Health, Tartu, Estonia

## Abstract

**Background:**

Objectives of the study were to document the impact of some management factors on the occurrence of clinical mastitis in primiparous dairy cows and to identify common udder pathogens of clinical mastitis in freshly calved heifers and multiparous cows on the day of calving.

**Methods:**

A one-year study was conducted during 2004 and 2005 in 11 selected Estonian dairy herds. Data consisted of 68 heifers with clinical mastitis and 995 heifers without clinical mastitis on the day of calving. Multivariable logistic regression with a random herd effect was used to investigate any association between housing system or the time interval from movement of heifers to the calving facility and day of calving on occurrence of clinical mastitis. Milk samples for bacteriological analysis were collected from affected heifers and multiparous cows on the day of calving

**Results:**

Clinical mastitis occurrence in the study population of freshly calved heifers equalled 6.1 %. Housing system was not a significant risk factor for clinical mastitis of freshly calved heifers.

Moving heifers to the cowbarn less than two weeks before calving in tiestall farms increased risk (OR = 5.9 p = 0.001) for clinical mastitis at parturition. The most frequently isolated udder pathogens among heifers were *Escherichia coli *(22.1%), *Streptococcus uberis *(19.1%) and coagulase-negative staphylococci (8.8%). In comparison, the main pathogen in multiparous cows with clinical mastitis at parturition was *Staphylococcus aureus *(11.2%).

**Conclusion:**

Moving heifers to the calving facilities too late in tiestall farms increased risk for clinical mastitis at parturition. The isolated udder pathogens did not differ significantly in tiestall farms compared to freestall farms in heifers, but differences were found between heifers and multiparous cows at parturition.

## Background

Mastitis is an economically important disease for dairy cattle production worldwide. Although replacement heifers are generally expected to have good udder health, many studies have identified a high risk of their developing subclinical mastitis during early lactation and reported that the prevalence of intramammary infections (IMI) is high in the peripartum period [1-7], mainly depending on infectious species [8]. At the same time, published reports on clinical mastitis incidence in freshly calved heifers are scarce and controversial. A nested case-control study in Norway showed that 5 % (6,410 out of 128,027) cases of clinical mastitis was treated in first calving heifers [9]. In Finland, the frequency of treatments for heifer mastitis from one week before to one week after calving was 3.9% for Ayrshires and 5.6% for Frisians [10]. In a study conducted in Netherlands the rate of clinical mastitis around parturition was found to be higher in heifers (>30%) compared to older cows (13%) [11].

Coagulase negative staphylococci (CNS), *Streptococcus dysgalactiae *(*Str. dysgalactiae*) and coliforms have been the most commonly identified pathogens of clinical mastitis during the periparturient period in heifers [12,13]. However, in the studies conducted in Norway, *Staphylococcus aureus (S. aureus*) was the most frequently isolated micro-organism, followed by *Str. dysgalacatiae *and CNS [14]. At the same time, differences have been found in occurrence of staphylococcal mastitis between primiparous and multiparous cows, where CNS was more prevalent among cows and *S. aureus *in freshly calved heifers [15]. Some studies have suggested that udder pathogens found in heifers close to parturition are similar to mammary pathogens found in lactating cows [1,12]. On the other hand, the risk of *S. aureus *IMI was influenced by the amount of time the heifers were housed with older cows and by the proportion of *S. aureus- *infected cows in the herd [16]. In Estonia, the most common pathogens of clinical mastitis are *S. aureus *(20.5% of isolated bacteria), CNS (11%), *Streptococcus agalactiae *(*Str. agalactiae*) (10.7%) and *Streptococcus uberis *(*Str. uberis*) (10.5%) [17]. No data are available on udder health in freshly calved heifers and multiparous cows in Estonia, although clinical mastitis has frequently been observed at parturition. Management factors at the herd level, including housing, feeding and milking systems, affect the incidence of clinical mastitis [18-21]; whereas at the individual cow level, milk leakage, teat and udder oedema and blood in the milk are associated with mastitis incidence [22]. Both types of associations are dependent upon species of udder pathogens that are present [23]. The transition phase, typically defined as the period from 3 weeks before to 3 weeks after parturition, is viewed as a critical time in the lactation cycle of a dairy cow. During this period, the cow experiences a series of nutritional, physiological and social changes which render her more vulnerable to infectious and metabolic diseases [24].

The aims of this study were:

1) to study whether mastitis occurrence in first calving heifers differs between farms with different housing systems and whether it is affected by the time interval between movement of heifers to their calving facility and their day of calving.

2) to identify common udder pathogens of clinical mastitis in first-calving heifers and multiparous cows on the day of calving in Estonia

## Methods

### Study population and experimental design

The one year study was carried out during 2004 and 2005. Eleven large-scale Estonian dairy herds was used in this study. These herds were selected from among the herds who received regular herd health visits by the university large animal clinic (in total 25 herds). The herds having more than 100 cows and 50 replacement heifers calving per year were included into the study. In Table 1, the main characteristics of the selected herds are presented. All heifers that calved during the observation period (n = 1,063) were eligible for inclusion. Heifers with clinical mastitis on the day of calving were included as cases (n = 68), and the remaining freshly calved heifers (n = 995) were controls. Heifers on each farm were moved from their rearing facility to the milking farm according to the availability of space. The number of days between the day of transfer of the heifer to the cowshed and the day of calving was recorded.

**Table 1:** Characteristics of farms used in the study

	**Tied housing**	**Loose housing**
Number of herds	6	5
Average herd size (min; max)	259(200–350)	318(130–460)
Average milk yield per herd kg/305 d (min; max)	8056(5822–9130)	7194(6206–8061)
Total number of freshly calved heifers	423	640
Average number of calved heifers per herd (min; max)	71(50–82)	128(50–270)

### Data collection in cases of clinical mastitis

Local trained veterinarians collected milk samples during the first milking from all freshly calved heifers and multiparous cows on the day of calving. If milk from a quarter had abnormal viscosity (watery, thicker than normal), color(yellow, blood-tinged) or consistency(flakes or clots), clinical mastitis was diagnosed, and samples from diseased udder quaters were collected for bacteriological examination [25]. Before collection, the teat end was cleaned with 70%-ethanol swabs and allowed to dry. After discarding a few streams of milk, samples (2 to 4 ml) were collected into sterile 10 ml plastic tubes, either frozen at -20°C or cooled to +4°C and transported to the Estonian Veterinary and Food Laboratory. All bacteriological examinations of milk samples were performed according to the standards of the National Mastitis Council [26].

### Data analysis

Logistic regression with a random herd effect for controlling clustering was used to analyze the effect of housing system (freestall, tiestall with short stall-length or tiestall with long stall-length) and length of time before calving that the heifers had been moved to the calving facility on the occurrence of clinical mastitis. To simplify the modelling, the continuous variable, number of days from moving heifers to the calving facility and expected parturition, was transformed to a dichotomous variable (≤14 days vs. >14 days classes) in the model. Odds ratios (OR) with a 95% confidence interval (95% CI) were calculated. Statistical significance was assumed at p ≤ 0.005. These analyses were conducted using Stata 9.2 [27]. A two-sample proportion test was used to estimate statistical significance of differences in occurrence of udder pathogens between first-calving heifers and multiparous cows. These analyses were conducted using statistical software Statistix for Windows 2.0.

## Results

Approximately 40% (423) of the first-calving heifers were in tiestall farms and approximately 60% (640) were in freestall farms. The overall occurrence of clinical mastitis at calving of the heifers was 6.4% (n = 68), being 9.7% (n = 41) in tiestall farms compared with 4.1 % (n = 27) in freestall farms. The range of days from moving heifers to the calving facility and expected parturition were from 0 to76, where the median day was 26. The results of logistic regression analysis are shown in Table 2. Housing system only was not a significant risk factor for clinical mastitis of freshly calved heifers. In tiestall farms heifers moved to the calving facility less than two weeks before expected parturition had a higher risk (OR = 5.9 p = 0.001) to develop clinical mastitis at calving than heifers moved more than 14 days before calving.

**Table 2:** Summary of logistic modelling of risk factors for clinical mastitis in heifers on the day of calving in eleven Estonian dairy herds.

Risk factor	Number of cases(n = 68)	Number of controls(n = 995)	OR^1^	95% CI OR^2^	P-value
Model 1					
Tiestall, short stall-length (≤ 175 cm), vs. tiestall, long stall-length (> 175 cm)	27/14	214/168	2.12	0.32–14.2	0.43
Freestall vs.tiestall, long stall-length	27/14	613/168	0.60	0.09–3.75	0.58

Model 2					
Freestall	27	613	0.39	0.85–1.83	0.237
Tiestall	41	382	1		
>14 day between movement to calving facility and day of calving	32	419	3.39	1.42–8.07	0.006
>14 days between movement to calving facility and day of calving	36	576	1		

Tiestall and >14 days	16	260	1		
Tiestall and ≤14 days	25	122	5.91	1.98–17.66	0.001
Freestall and >14 days	20	284	0.78	0.13–4.57	0.79
Freestall and ≤14 day	7	329	1.08	0.16–7.05	0.94

^1 ^Odds ratio

^2 ^95% confidence interval odds ratio

In total, 303 clinical mastitis cases were identified on the day of parturition in 2,355 multiparous cows (12.8%). Udder pathogens were isolated from 49 (72%) out of 68 cases of clinical mastitis in freshly calved heifers and from 185 (61%) out of 303 cases in multiparous cows.

Bacteriological findings are shown in Table 3. The most frequently isolated bacteria from milk samples of freshly calved heifers were *E.coli *and *Str. uberis*. No clinical mastitis caused by *Str. agalactiae *or *Corynebacterium spp*. was discovered, and only one case of *S. aureus *mastitis was found in heifers. In contrast, *S. aureus *was the most common bacterium isolated from milk of affected multiparous cows, followed by *Str. uberis *and Escherichia coli(*E. coli*). Occurrence differences between heifers and cows were statistically significant for *Str. uberis *(p = 0.037), coliforms (p = 0.0002) and *S. aureus *(p = 0.019).

**Table 3:** Bacterial species isolated from milk samples from heifers and multiparous cows having clinical mastitis at parturition

	**Heifers**		**Cows**	
**Pathogens**	%	n	%	n
	
E.coli*	22.1	15	6.6	20
Str. uberis*	19.1	13	9.9	30
CNS	8.8	6	7.3	22
*Lactococcus lactis*	4.4	3	5.0	15
*Klebisella spp*.	4.4	3	2.3	7
*Str. spp*	2.9	2	3	9
*Enterococcus spp*	2.9	2	2.3	7
*Pseudomonas spp*	2.9	2	0.7	2
S.aureus*	1.5	1	11.2	34
*Arcanobacterium spp*	1.5	1	2.6	8
*Str.dysgalactiae*	1.5	1	3.6	11
*Corynebacterium spp*	0	0	2.0	6
*Str. agalactiae*	0	0	3.3	10
*Candida spp*	0	0	1.3	4
No growth	25	17	29.4	89
Mixed culture	2.9	2	9.6	29
Total	100.0	68	100.0	303

* The difference between heifers and multiparous cows is statistically significant (

**Figure 1:**
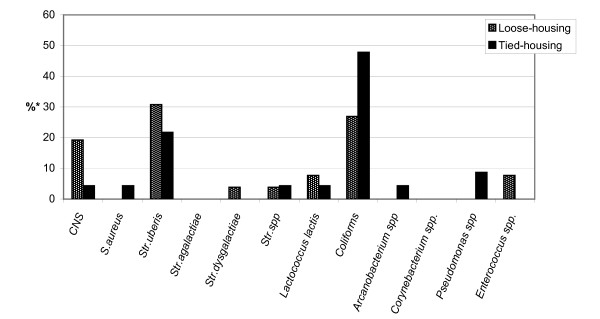
Distribution of udder pathogens in freshly calved heifers in two housing systems. Detailed legend: *calculated against the total number of isolates from heifers of each housing system.

Figure 1 shows the distribution in tiestall vs. freestall housing systems of udder pathogens isolated from quarter milk samples with clinical mastitis in freshly calved heifers.

In tiestall herds, 36.6% (n = 15) of the samples were bacteriologically negative or mixed cultures, while the corresponding proportion in freestall herds was 14.8% (n = 4). The most common udder pathogens in both housing systems were *Str. uberis, E. coli *and CNS. Occurrence of colimastitis was higher in freestall farms than in tiestall farms, but *Str. uberis *was more frequent in tiestall farms than freestall farms. The differences were not statistically significant.

## Discussion

In 11 large herds using traditional Estonian dairy management, two housing systems did not differ significantly in clinical mastitis occurrence of first-calving heifers. Others, however, have reported higher incidence of clinical mastitis in tiestall than in freestall housing [21,28-30]. In tiestall farms, the main risk factors for clinical mastitis are teat injuries, short stalls and shortage of bedding material [31,32], especially during the periparturient period [33]. In one Swedish report, the incidence of clinical mastitis decreased across 18 months, after the management system was changed from the tiestall to the freestall system [34]. We did identify an association at the tie-stall farms between time of movement of close-to-term heifers to the milking farm and the occurrence of clinical mastitis. Stress and sudden changes in environmental and management conditions during the peripartum period could weaken natural defence mechanisms in animals, making them more susceptible to clinical mastitis. In tiestall systems, an increased frequency of lying down and rising may lead to increased risk of teat tramping, leading to increased clinical mastitis incidence [35]. Contrarily, in loose-housing systems, cows have sufficient space for lying down and standing up in a more natural way during parturition. The results of the present study reflect the situation in large commercial dairy herds in Estonia. However, the number of herds in the study was limited and because sample sizes were small in some herds, these results should be interpreted with caution. A larger study of longer duration and with more herds is needed for more reliable conclusions.

In the relatively few reports on clinical mastitis in heifers, occurrence of clinical mastitis has been variable. In Finnish studies by Myllys [10], the treatment of clinical mastitis in heifers from one week before through one week after calving increased from 1.8% to 4.4% between 1983 and 1991. In the USA, the incidence of clinical mastitis in heifers was 12.3%, and mostly coliforms and streptococci were isolated [36]. In 1,040 heifers, 1361 clinically affected quarters were found in a large-scale Norwegian study [14]. As to the present investigation, the occurrence of clinical mastitis in freshly calved heifers was a modest 6.1%.

Environmental bacteria dominated in our study. Mainly *E.coli *(22.1%), *Str.uberis *(19.1%) and CNS (9.2%) were isolated in cases of clinical mastitis of the freshly calved heifers. Similar results have been reported by others, in which common bacteria isolated after parturition were CNS, coliforms and streptococci [12,13,36].

In a Danish study, the most frequently isolated organism was *S. aureus *[14]. Our investigation did not show *S. aureus *clinical mastitis in freshly calved heifers, although *S. aureus *was the main pathogen among the multiparous cows. Despite that, the spread of this infection should not be underestimated. Comparing tiestall and freestall farms, the bacterial findings on the day of parturition were generally the same. Coliform infection was more common among loose-housed heifers, where the primary source of infection is bovine faeces and where the secondary multiplication of bacteria to high numbers in bedding and manure is often a risk factor [38]. Prevalence of *Str. uberis *infections depends on udder and calving hygiene, but immune response in the lower udder gland also plays an important role [37]. That might explain the higher prevalence of clinical mastitis in heifers. Altough more CNS infections were found in tiestall farms, we could not draw clear conclusions due to the small number of samples. Our findings confirmed that *S. aureus *could be the main pathogen causing mastitis in multiparous cows at the time of parturition in Estonia. The importance of environmental bacteria may increase if management systems evolve towards higher intensity of production.

## Conclusion

Moving heifers to the calving fascilities too late in tiestall farms, increased risk for clinical mastitis at parturition. The isolated udder pathogens did not differ significantly in tiestall farms compared to freestall farms in heifers, but differences were found between heifers and multiparous cows at parturition.

## Competing interests

The author(s) declare that they have no competing interests.

## Authors' contributions

PK carried out the study, compiled the results and drafted the manuscript. AV participated in the designing the study and analysis of the data. BA coordinated data collection, and KK coordinated the study. All authors were significantly involved in designing the study, intepreting of data and composing the manuscript.
